# On the Metabolism of Exogenous Ketones in Humans

**DOI:** 10.3389/fphys.2017.00848

**Published:** 2017-10-30

**Authors:** Brianna J. Stubbs, Pete J. Cox, Rhys D. Evans, Peter Santer, Jack J. Miller, Olivia K. Faull, Snapper Magor-Elliott, Satoshi Hiyama, Matthew Stirling, Kieran Clarke

**Affiliations:** ^1^Department of Physiology, Anatomy and Genetics, University of Oxford, Oxford, United Kingdom; ^2^Clarendon Laboratory, Department of Physics, University of Oxford, Oxford, United Kingdom; ^3^NTT DOCOMO Inc., Yokosuka, Japan; ^4^Innovative Physical Organic Solutions (IPOS), University of Huddersfield, Huddersfield, United Kingdom

**Keywords:** (R)-3-hydroxybutyl (R)-3-hydroxybutyrate, ketone ester, ketone salt, ketones, d-β-hydroxybutyrate, exogenous ketones

## Abstract

**Background and aims:** Currently there is considerable interest in ketone metabolism owing to recently reported benefits of ketosis for human health. Traditionally, ketosis has been achieved by following a high-fat, low-carbohydrate “ketogenic” diet, but adherence to such diets can be difficult. An alternative way to increase blood D-β-hydroxybutyrate (D-βHB) concentrations is ketone drinks, but the metabolic effects of exogenous ketones are relatively unknown. Here, healthy human volunteers took part in three randomized metabolic studies of drinks containing a ketone ester (KE); (R)-3-hydroxybutyl (R)-3-hydroxybutyrate, or ketone salts (KS); sodium plus potassium βHB.

**Methods and Results:** In the first study, 15 participants consumed KE or KS drinks that delivered ~12 or ~24 g of βHB. Both drinks elevated blood D-βHB concentrations (D-βHB C_max_: KE 2.8 mM, KS 1.0 mM, *P* < 0.001), which returned to baseline within 3–4 h. KS drinks were found to contain 50% of the L-βHB isoform, which remained elevated in blood for over 8 h, but was not detectable after 24 h. Urinary excretion of both D-βHB and L-βHB was <1.5% of the total βHB ingested and was in proportion to the blood AUC. D-βHB, but not L-βHB, was slowly converted to breath acetone. The KE drink decreased blood pH by 0.10 and the KS drink increased urinary pH from 5.7 to 8.5. In the second study, the effect of a meal before a KE drink on blood D-βHB concentrations was determined in 16 participants. Food lowered blood D-βHB C_max_ by 33% (Fed 2.2 mM, Fasted 3.3 mM, *P* < 0.001), but did not alter acetoacetate or breath acetone concentrations. All ketone drinks lowered blood glucose, free fatty acid and triglyceride concentrations, and had similar effects on blood electrolytes, which remained normal. In the final study, participants were given KE over 9 h as three drinks (*n* = 12) or a continuous nasogastric infusion (*n* = 4) to maintain blood D-βHB concentrations greater than 1 mM. Both drinks and infusions gave identical D-βHB AUC of 1.3–1.4 moles.min.

**Conclusion:** We conclude that exogenous ketone drinks are a practical, efficacious way to achieve ketosis.

## Introduction

Human's ability to produce and oxidize ketone bodies arguably evolved to enhance survival during starvation by providing an energy source for the brain and slowing the breakdown of carbohydrate and protein stores (Owen et al., 1967; Sato et al., 1995; Marshall, 2010). The brain is normally reliant on carbohydrate as a substrate, being less able to metabolize lipids, despite adipose tissue representing a far larger energy store than muscle and liver glycogen. Therefore, during starvation, lipids are used for hepatic ketogenesis and, via ketone bodies, lipids sustain the brain. Endogenous production of the ketone bodies, d-β-hydroxybutyrate (βHB) and acetoacetate (AcAc), increases slowly, driven by interactions between macronutrient availability (i.e., low glucose and high free fatty acids) and hormonal signaling (i.e., low insulin, high glucagon and cortisol). Produced continuously under physiological conditions, blood ketone concentrations increase during starvation (Cahill, 1970), when consuming a “ketogenic” (low carbohydrate, high-fat) diet (Gilbert et al., 2000) or following prolonged exercise (Koeslag et al., 1980).

Ketogenic diets have been successfully used to treat diseases that have an underlying metabolic component, effectively decreasing seizures in recalcitrant pediatric epilepsy (Kossoff et al., 2003), lowering blood glucose concentrations in type 2 diabetes mellitus (Feinman et al., 2015) and aiding weight-loss (Bueno et al., 2013). Emerging evidence supports several clinical uses of ketogenic diets, for example in neurodegenerative diseases (Vanitallie et al., 2005), specific genetic disorders of metabolism (Veech, 2004) and as an adjunct to cancer therapy (Nebeling et al., 1995). Ketone bodies themselves may underlie the efficacy of the ketogenic diet, either through their role as a respiratory fuel, by altering the use of carbohydrate, protein and lipids (Thompson and Wu, 1991; Cox et al., 2016), or through other extra- and intracellular signaling effects (Newman and Verdin, 2014). Furthermore, ketone metabolism may offer a strategy to improve endurance performance and recovery from exercise (Cox et al., 2016; Evans et al., 2017; Holdsworth et al., 2017; Vandoorne et al., 2017). However, achieving compliance to a ketogenic diet can be difficult for both patients and athletes and may have undesirable side effects, such as gastro-intestinal upset (Cai et al., 2017), dyslipidemia (Kwiterovich et al., 2003) or decreased exercise “efficiency” (Edwards et al., 2011; Burke et al., 2016). Hence, alternative methods to raise blood ketone concentrations have been sought to provide the benefits of a ketogenic diet with no other dietary changes.

An alternative to the ketogenic diet is consumption of drinks containing exogenous dietary ketones, such as ketone esters (KE) and ketone salts (KS). The metabolic effects of KS ingestion have been reported in rats (Ari et al., 2016; Kesl et al., 2016; Caminhotto et al., 2017), in three extremely ill pediatric patients (Plecko et al., 2002; Van Hove et al., 2003; Valayannopoulos et al., 2011) and in cyclists (O'Malley et al., 2017; Rodger et al., 2017). However, the concentrations of blood βHB reached were low (<1 mM) and a high amount of salt, consumed as sodium, potassium and/or calcium βHB, was required to achieve ketosis. Furthermore, dietary KS are often racemic mixtures of the two optical isoforms of βHB, d-βHB, and l-βHB, despite the metabolism of l-βHB being poorly understood (Webber and Edmond, 1977; Scofield et al., 1982; Lincoln et al., 1987; Desrochers et al., 1992). The pharmacokinetics and pharmacodynamics of KS ingestion in healthy humans at rest have not been reported.

Ketone monoester and diester compounds may circumvent the problems associated with inorganic ion consumption in KS drinks. KE ingestion rapidly increased blood ketone concentrations to >5 mM in animals (Desrochers et al., 1995a,b; Clarke et al., 2012a) and the first oral, non-racemic KE for human consumption, (R)-3-hydroxybutyl (R)-3-hydroxybutyrate, raised blood βHB concentrations to 3–5 mM in healthy adults (Clarke et al., 2012b; Shivva et al., 2016) and athletes (Cox et al., 2016; Holdsworth et al., 2017; Vandoorne et al., 2017). However, the pharmacokinetics and pharmacodynamics of this KE with confounding factors, such as prandial state or multiple KE drinks, have not been characterized.

Here we investigated the effects of KE and KS consumption on blood βHB and metabolite concentrations. As we found that KE ingestion delivered a >50% higher plasma concentrations of d-βHB alone, we subsequently determined the reliability and repeatability of ketosis following KE consumption and the effects of concomitant meal ingestion on blood ketone and substrate kinetics. Finally, we determined whether nasogastric infusion could be used for KE administration, given that some patients require feeding in this manner.

## Materials and methods

### Study design

Firstly, in a randomized four-arm cross-over study, blood βHB concentrations were compared following ingestion of equal amounts of βHB as a KE or a KS at two doses by healthy volunteers at rest (Study 1; *n* = 15). Secondly, in a randomized five-arm cross-over study, inter- and intra-participant repeatability of ketosis was examined following ingestion of identical KE drinks, twice whilst fed and twice whilst fasted. As a control, participants also consumed one isocaloric (1.9 kCal.kg^−1^) dextrose drink (Study 2; *n* = 16). Finally, blood d-βHB was measured after equal amounts of KE were given as three drinks (*n* = 12) or a constant nasogastric (NG) infusion (*n* = 4) (Study 3; total *n* = 14) over 9 h.

### Participants and screening

These studies were approved by external Research Ethics Committees (London Queen's Square: 14/LO/0288 and South West Frenchay: 15/SW/0244) and were conducted in accordance with the Declaration of Helsinki (2008). Studies took place at the University of Oxford between September 2014 and September 2016. Participants were healthy, aged 21–57, non-smokers and had no history of major illness. Female participants were using oral contraception to minimize the effects of menstrual phase on results. Participants provided written informed consent prior to inclusion, and completed a confidential medical screening questionnaire to determine eligibility. Anthropometric characteristics are shown in Table 1. Sample sizes were chosen following an estimated power calculation based on the effect size in previous work using KE drinks (Clarke et al., 2012b; Shivva et al., 2016).

**Table 1:** Physical characteristics of subjects.

**Characteristic**	**Study 1 (*n* = 15)**	**Study 2 (*n* = 16)**	**Study 3 (*n* = 14)**
	**Mean (*SD*)**	**Mean (*SD*)**	**Mean (*SD*)**
Age (y)	22.7 (2.1)	27.4 (6.5)	30.2 (10.1)
Height (m)	1.76 (0.10)	1.80 (0.10)	1.77 (0.10)
Weight (kg)	70.5 (11.1)	72.7 (15.4)	70.4 (11.2)
BMI (kg/m^2^)	22.7 (2.2)	22.5 (2.5)	22.4 (2.5)
M/F	9/6	10/6	9/5

### General visit procedures

Participants refrained from alcohol and caffeine for 24 h prior to each visit AND were asked to consume a similar meal the night before each visit. All studies were carried out at the University of Oxford Human Physiology Laboratories and started at 0800 h following an overnight (>8 h) fast, with a minimum of 72 h between visits. Visit order was randomized prior to commencement by an administrative investigator using a pseudo-random number generator to produce a list of combinations of visit order, which were then allocated based on order of enrolment by a different investigator.

Fasting blood samples were collected prior to all interventions. Following consumption of study drinks (details below), blood, expired gas and urine samples were collected at regular intervals for 4 h. Water was freely permitted and participants remained sedentary at the test facility throughout the visit. A subset of participants returned for samples 8 and 24 h after the ketone drinks (Study 1).

### Blood sampling and analysis

Venous blood samples (2 ml) were obtained during all visits using a 22 G catheter inserted percutaneously into an antecubital vein. The catheter was kept patent using a saline flush following each sample collection. Additionally, during Study 1, arterialized blood from a catheter inserted into a heated hand (Forster et al., 1972) was collected into heparinized blood gas syringes (PICO 100, Radiometer, Copenhagen) from a subset of participants (*n* = 7) and immediately analyzed for pH and electrolytes using a clinical blood gas analyser (ABL, Radiometer, Copenhagen).

d-βHB was measured immediately on whole blood using a handheld monitor and enzyme-based reagent strips (Precision Xtra, Abbott Diabetes Care, UK). Samples were stored on ice, centrifuged and duplicate plasma aliquots stored at −80°C. All urine passed during the visit was collected, the total volume recorded, and 1 ml aliquots taken, frozen and retained for analysis.

Plasma glucose, free fatty acids (FFA), triglycerides (TG) and urinary d-βHB were assayed using a commercial semi-automated bench-top analyzer (ABX Pentra, Montpellier, France), and insulin was measured using a commercially available ELISA assay (Mercodia, Uppsala, Sweden). Both the pure liquid KS and KE, and a subset of plasma (*n* = 5) and urine (*n* = 10) samples from a subset of participants in Study 1 underwent analysis using GC-MS and a chiral column, and the concentrations of l-βHB was calculated using the enzymatically determined concentration of d-βHB and the ratio of the d/l-βHB peaks obtained through GC-MS. Acetoacetate was assayed using an enzymatic method (Bergmeyer, 1965), and breath acetone was measured using GC-MS (Study 1) or with a handheld electrochemical device (Study 2; NTT DOCOMO, Japan) (Toyooka et al., 2013).

### Study protocols

#### Study 1

Over four visits, participants (*n* = 15) consumed 1.6 and 3.2 mmol.kg^−1^ of βHB as KE (141 mg/kg and 282 mg/kg of R-3-hydroxybutyl-R-1,3-hydroxybutyrate) or as KS (KetoForce, KetoSports, USA) sodium and potassium βHB, containing 1.6–3.2 g of each cation), plus 6 g of sweetener containing 19 kCal (4 g of carbohydrate) (Symrise, Holzminden, Germany), diluted to 300 ml using water. Drink blinding was not possible due to unmaskable differences in taste (bitter vs. salty).

#### Study 2

Over five visits, participants (*n* = 16) consumed either 4.4 mmol.kg^−1^ of βHB (2.2 mmol.kg^−1^ or 395 mg/kg of KE; 1 mole of KE delivered 2 moles of d-βHB equivalents): twice whilst fasted, and twice following a standardized meal, or an isocaloric dextrose drink without a meal. To improve palatability, drinks were diluted to 500 ml with a commercially available, citrus flavored drink containing 65 kCal (5 g of carbohydrate) (Glaceau, UK). The dextrose drink was taste-matched using a bitterness additive (Symrise, Holzminden, Germany). The standard meal consisted of porridge oats (54 g), semi-skimmed milk (360 ml) and banana (120 g), giving 600 kCal per person, with a macronutrient ratio of Carbohydrate: Protein: Fat of 2:1:1.

#### Study 3

Participants consumed 13.2 mmol.kg^−1^ of βHB (6.6 mmol.kg^−1^ or 1,161 mg/kg of KE) over 9 h, either as 3 drinks of 4.4 mmol.kg^−1^ of βHB at 3 h intervals (*n* = 12), or as an initial bolus of 4.4 mmol.kg^−1^ of βHB given through a nasogastric tube, followed by an infusion of 1.1 mmol.kg.h^−1^, beginning 60 min after the initial bolus, for 8 h (*n* = 4). Two participants completed both conditions (total *n* = 14). In both conditions, the KE was diluted to 1.5 L using the same citrus water as used in Study 2.

### Statistical methods

For all studies, the area under the curve (AUC) of blood [βHB] was calculated using the trapezium rule. In Study 3, for each of the three drinks, the initial rate of d-βHB appearance was estimated using d-βHB concentrations at baseline and 30 min post-drink, and d-βHB elimination was estimated using the AUC between the post-drink peak (60 min) and trough (180 min) d-βHB concentrations, with a baseline correction to the value at 180 min.

Unless otherwise stated, statistical analysis was conducted using Prism 6™ software. Values, expressed as means ± SEM, were considered significantly different at *p* < 0.05. Initial tests were undertaken to ensure that normality and sphericity assumptions were not violated. Subsequently, either one or two way repeated measures ANOVA, or Freidman's test with *post-hoc* Tukey or Dunnet's correction were performed, to compare changing concentrations of substrates, electrolytes, pH, insulin, breath and urinary βHB: both over time and between study interventions. In Study 2, data from each of the two study visits in each condition (fed and fasted) completed by an individual were included in the analysis.

In Study 2 a Student's unequal variance *t*-test with equal SD was used to compare urine βHB concentrations. Additionally, a linear mixed effects model was constructed to estimate partitions of variance in R, using the lme4 and blme packages (Chung et al., 2013; Bates et al., 2015). Feeding state and visit number were fixed effects in this model, and inter-participant variability was a random effect. Inter-participant variability was calculated according to the adjusted generalized R^2^ metric (as proposed by Nakagawa and Schielzeth, 2013), to partition variance between the fixed effects of feeding, inter-participant variability, and residual variability. The coefficient of variation for βHB C_max_ and AUC were calculated using the method of Vangel (1996).

## Results

### Effect of KE and KS drinks on blood ketone bodies and substrates (Study 1)

**Figure 1:**
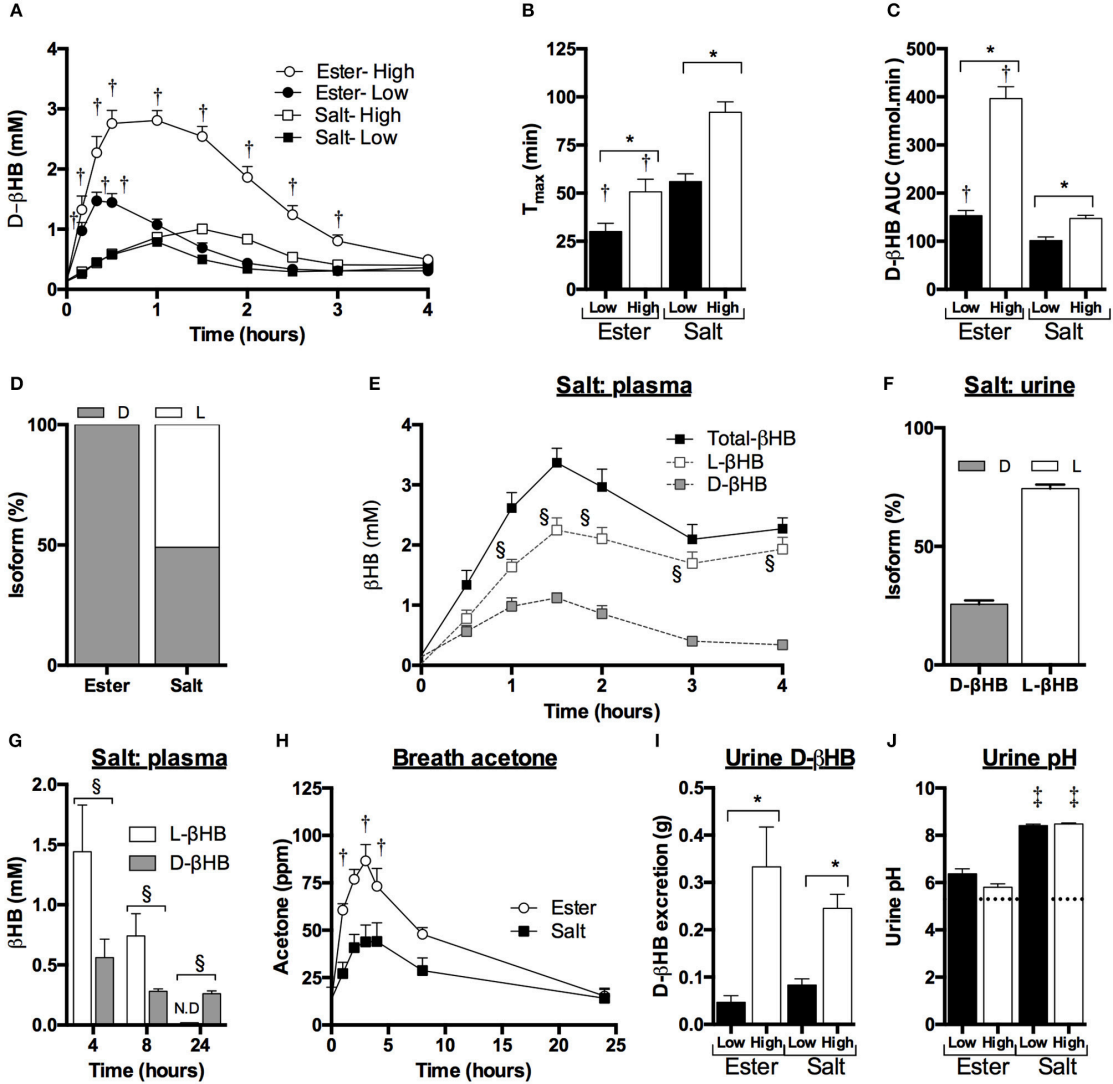
Blood, breath, and urine ketone kinetics following mole-matched ketone ester (KE) and ketone salt (KS) drinks, at two amounts, in 15 subjects at rest. Values are means ± SEM. **(A)** Blood d-βHB. **(B)** T_max_ of blood d-βHB. **(C)** AUC of blood d-βHB. **(D)** Isotopic abundance (%) of d- and l-chiral centers in pure liquid KE and KS. **(E)** Blood d-βHB and l-βHB concentrations in subjects (*n* = 5) consuming 3.2 mmol.kg^−1^ of βHB in KS drinks. **(F)** d-βHB and l-βHB concentrations in urine samples from subjects (*n* = 10) consuming 3.2 mmol.kg^−1^ of βHB in KS drinks. **(G)** Blood d- and l-βHB after 4, 8, and 24 h in subjects (*n* = 5) consuming 3.2 mmol.kg^−1^ of βHB in KS drinks. **(H)** Breath acetone over 24 h in subjects (n = 5) consuming 3.2 mmol.kg^−1^ of βHB in KE and KS drinks (ppm = parts per million). **(I)** Urine d-βHB excreted over 4 h after KE and KS drinks (*n* = 15). **(J)** Urine pH 4 h after drink, dotted line indicates baseline. ^†^*p* < 0.05 KE vs. equivalent amount of KS, ^*^p < 0.05 difference between 1.6 vs. 3.2 mmol.kg^−1^ of βHB, §*p* < 0.05 difference between amounts of d- and l-βHB, *p* < 0.05 difference between baseline and post-drink level.

Blood d-βHB concentrations rapidly increased to a maximum of 2.8 ± 0.2 mM following the KE drink and to 1.0 ± 0.1 mM following the KS drink (Figure 1A). After the peak was reached, blood d-βHB disappearance was non-linear, and followed first order elimination kinetics as reported previously (Clarke et al., 2012b; Shivva et al., 2016). d-βHB T_max_ was ~2-fold longer following KS drinks vs. KE drinks (*p* < 0.01, Figure 1B), and KS d-βHB AUC was ~30–60% lower than the KE drink (*p* < 0.01, Figure 1C).

To determine the reason for the differences in blood d-βHB concentration, the KE and KS drinks were analyzed for enantiomeric purity. The KE contained >99% of the d-isoform, whereas ~50% of the KS βHB was the l-isoform (Figure 1D). Plasma samples from participants who consumed the high dose KS drink (*n* = 5) were analyzed to reveal higher l-βHB than d-βHB, the total βHB C_max_ being 3.4 ± 0.2 mM (Figure 1E), with a total βHB AUC of 549 ± 19 mmol.min. After 4 h, plasma l-βHB remained elevated at 1.9 ± 0.2 mM; differences in urinary excretion of the two isoforms could not explain this observation as both d- and l-βHB were excreted in proportion to their blood AUCs (Figure 1F). Therefore, in order to determine the time required for l-βHB elimination, a follow-up experiment was undertaken in which subjects (*n* = 5) consumed 3.2 mmol.kg^−1^ of βHB as KE and KS with hourly blood and breath sample collection up to 4 h, plus additional samples at 8 h and 24 h post-drink. l-βHB was found to be 1.1 ± 0.1 mM at 4 h, and 0.7 ± 0.2 mM after 8 h, but undetectable after 24 h (Figure 1G). Low amounts of d-βHB (0.3 ± 0.1 mM) were present at 24 h, presumably due to endogenous production. Both ketone drinks significantly increased breath acetone concentration, but at a slower rate than blood d-βHB, reaching a peak after 3 h that was twice as high following the KE (87 ± 9 ppm) than the KS (44 ± 10 ppm), suggesting that d-βHB was readily converted to acetone, but l-βHB was not (*p* < 0.005, Figure 1H).

For subjects completing the initial experiment (*n* = 15), the amount of d-βHB excreted in the urine increased with d-βHB intake, but was <1.5% of the total βHB ingested and was not different between matched doses of KE vs. KS (Figure 1I). There was no change in urine volume produced in different study conditions. Baseline urinary pH (5.7 ± 0.1) was unchanged by KE ingestion (pH 6.4 ± 0.2. *p* = 0.8 vs. baseline) but was significantly alkalinized by KS consumption (pH 8.5 ± 0.1. *p* < 0.001 vs. baseline) (Figure 1J).

**Figure 2:**
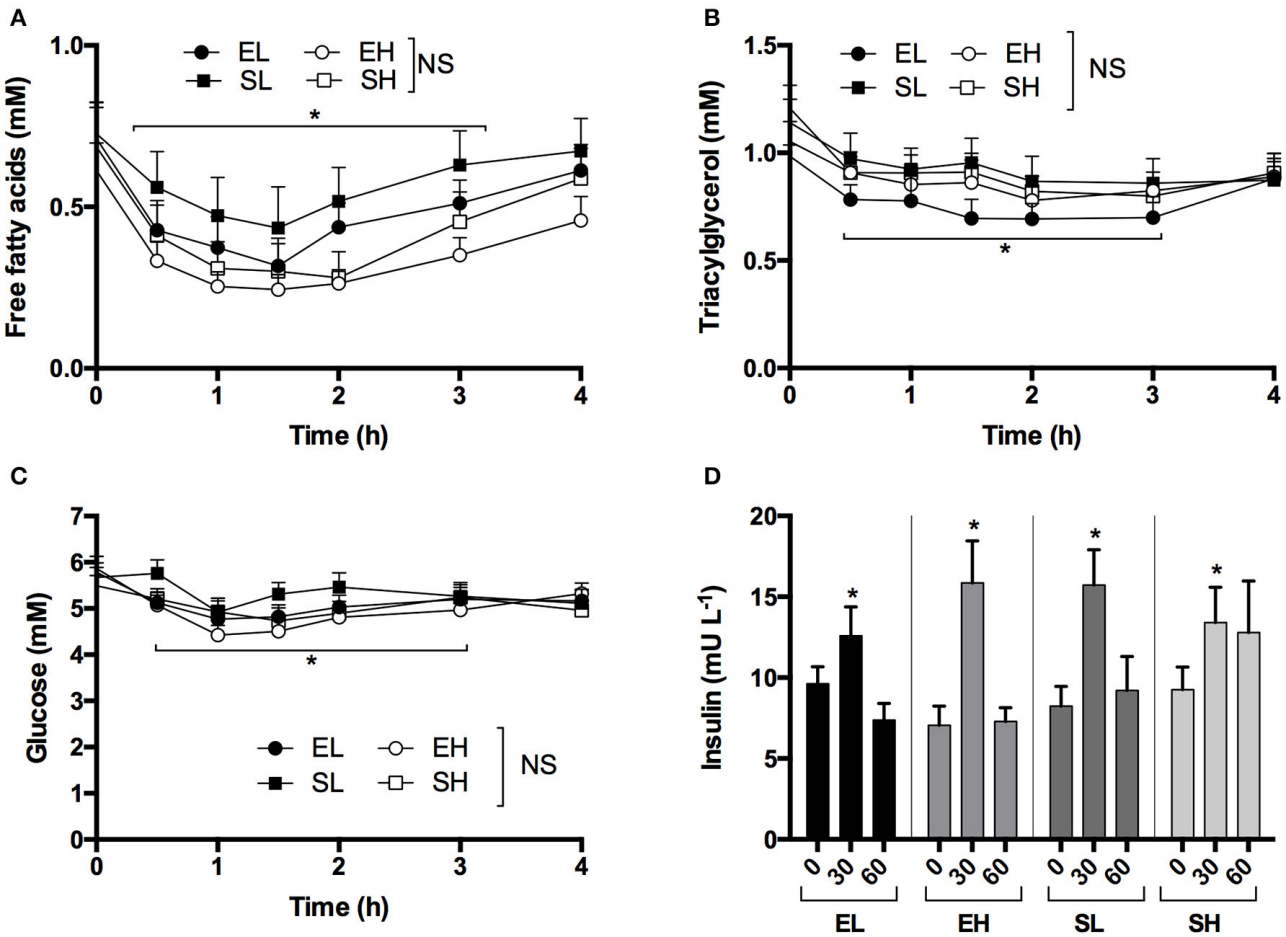
Concentrations of plasma non-esterified fatty acids, triacylglycerol, glucose, and insulin following equimolar ketone ester and ketone salt drinks, at two amounts, in subjects (*n* = 15) at rest. Values are means ± SEM. **(A)** Plasma FFA. **(B)** Plasma TG. **(C)** Plasma glucose. **(D)** Plasma insulin at baseline and after 30 and 60 min. EH, ketone ester high; EL, ketone ester low; SH, ketone salt high; SL, ketone salt low. ^*^p < 0.05 difference from baseline value.

Although decreases in FFA, TG and glucose occurred, there were no significant differences between the KE and KS drinks or with intake amount. Ingestion of ketone drinks significantly decreased overall mean plasma FFA from 0.7 to 0.4 mM, TG from 1.1 to 0.9 mM and glucose from 5.7 to 4.8 mM after 1 h (all *p* < 0.05). Concentrations were the same as at baseline by 4 h, with FFA at 0.6 mM, TG at 0.9 mM and glucose 5.1 mM (Figures 2A–C). There was a rise in insulin concentrations 30 min following all drinks, probably due to the small amount of carbohydrate in the sweetener (Figure 2D).

**Figure 3:**
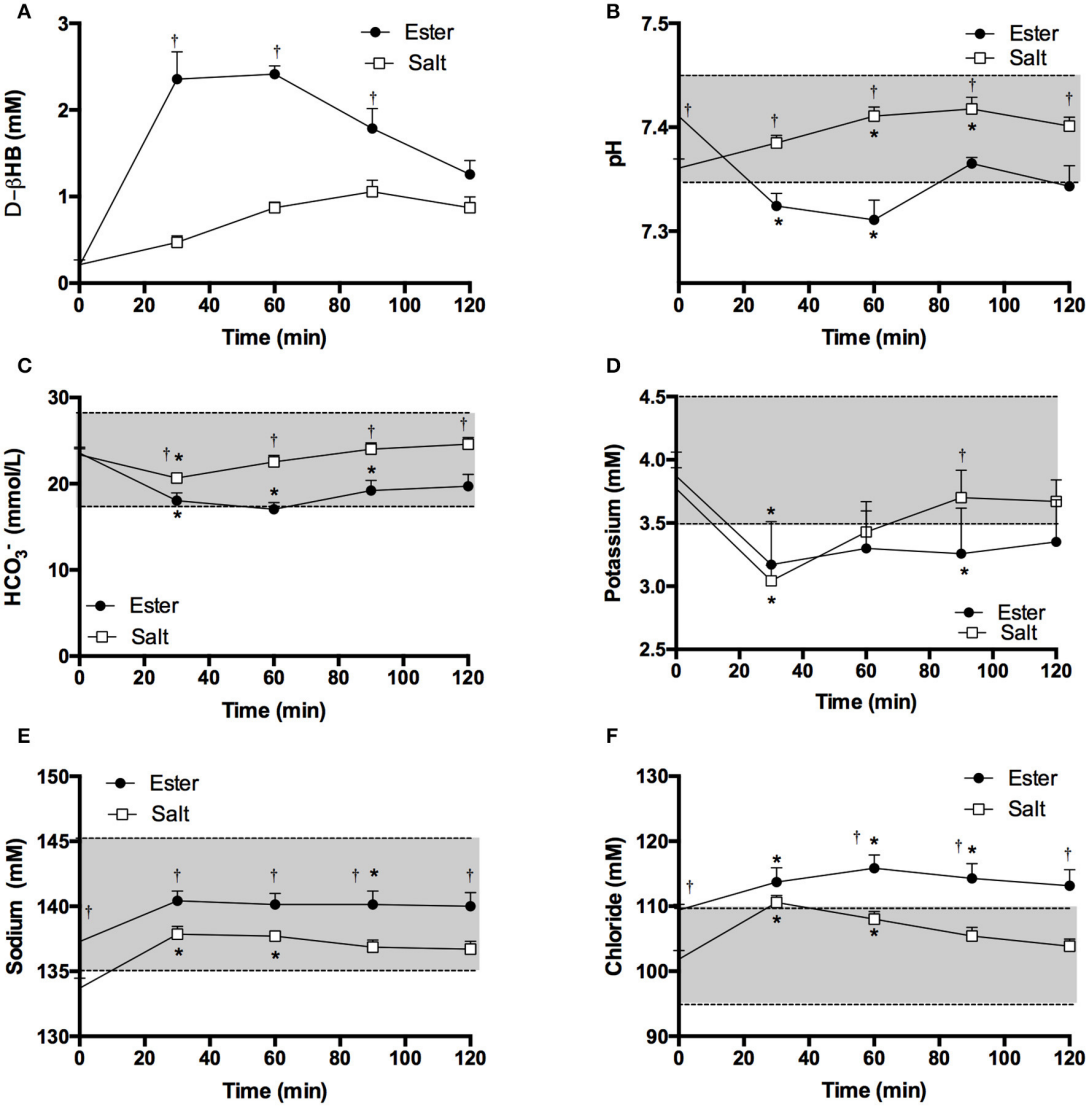
Blood d-βHB, pH, bicarbonate (${\text{HCO}}_{3}^{-}$) and electrolytes measured in arterialized blood samples from resting subjects (*n* = 7) following a ketone ester or salt drink containing 3.2 mmol.kg^−1^ of βHB. Shaded areas represent the normal range. Values are means ± SEM. **(A)** Venous blood d-βHB. **(B)** Arterialized blood pH. **(C)** Blood bicarbonate. **(D)** Blood potassium. **(E)** Blood sodium. **(F)** Blood chloride. ^†^*p* < 0.05 difference between KE and KS, ^*^p < 0.05 difference from baseline value.

In a subset of participants (*n* = 7) the effect of 3.2 mmol.kg^−1^ of βHB as KE and KS on blood pH and electrolytes after ketone drinks was investigated. Blood d-βHB kinetics were similar to those in the initial experiment (Figure 3A). After 60 min, blood pH declined from 7.41 to 7.31 following a KE drink (*p* < 0.001, Figure 3B). Bicarbonate fell significantly from 23.6 ± 0.7 to 17.0 ± 0.8 mM following KE drinks (*p* < 0.001), but remained within the normal range (Figure 3C). Both ketone drinks significantly decreased blood potassium concentrations by 0.7 mM (both drinks *p* < 0.05, Figure 3D) and increased sodium and chloride concentrations (Sodium: both drinks *p* < 0.05, Chloride: KE = *p* < 0.05, KS = *p* < 0.005, Figures 3E,F).

### Effect of meal consumption on blood d-βHB, repeatability of ketone kinetics and substrate changes following KE drinks (Study 2)

**Figure 4:**
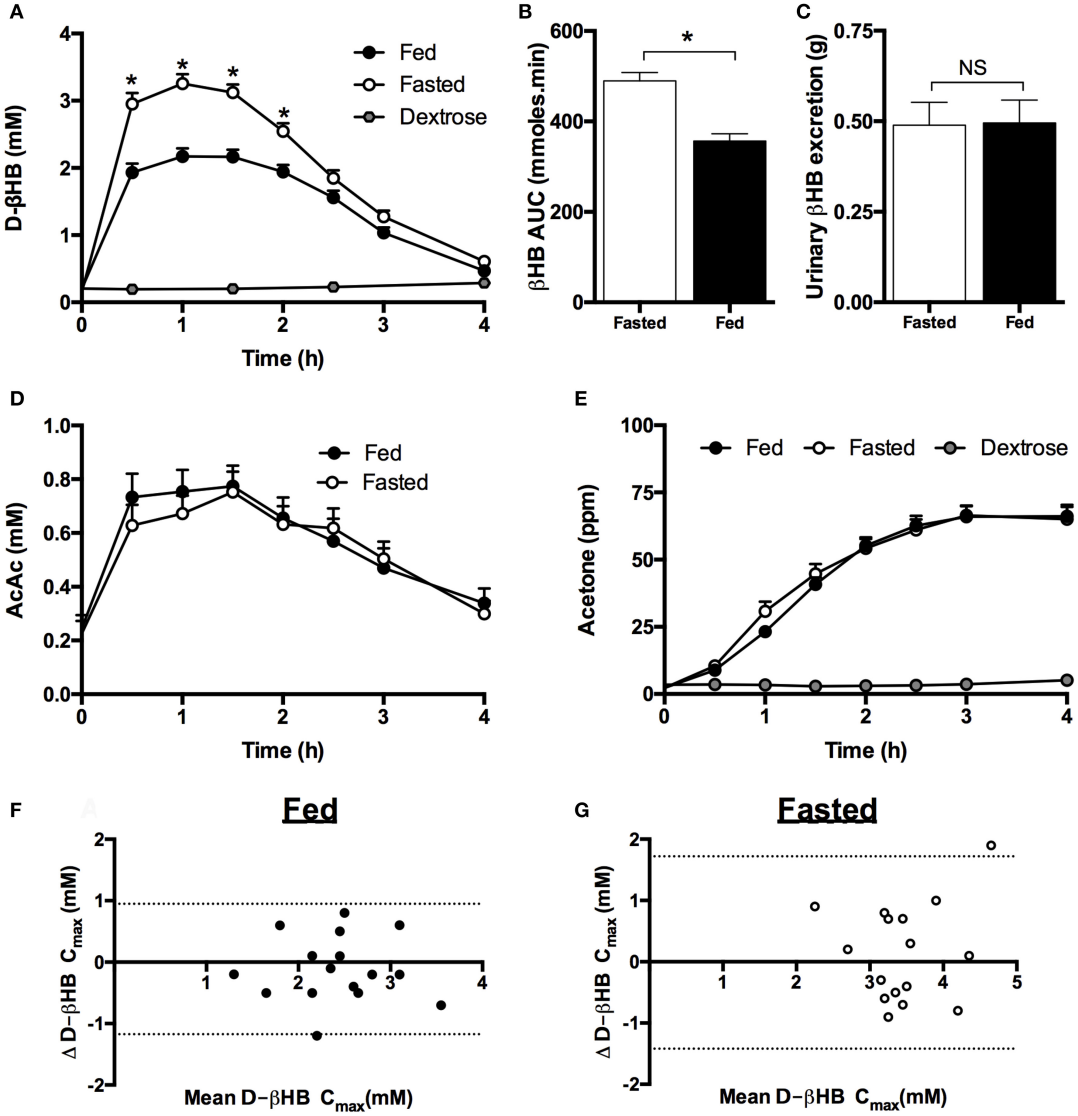
Blood, urine, plasma, and breath ketone concentrations following mole-matched ketone ester or isocaloric dextrose drinks in fed and fasted subjects (*n* = 16) at rest. Data from both of the two study visits in each condition (fed and fasted) completed by an individual are included in the analysis. Values are means ± SEM. **(A)** Blood d-βHB. **(B)** AUC of blood d-βHB. **(C)** Urine d-βHB excretion. **(D)** Plasma acetoacetate (AcAc). **(E)** Measured breath acetone (ppm = parts per million). **(F,G)** Mean d-βHB C_max_ and difference between βHB C_max_ over two visits when subjects separately consumed two ketone ester drinks in both the fed **(F)** and fasted **(G)** state. X axis = mean d-βHB C_max_ of the 2 visits (mM), Y axis = difference between d-βHB C_max_ in each visit. 95% confidence limits are shown as dotted lines. Significance denoted by: ^*^p < 0.05 fed vs. fasted.

A meal high in carbohydrate and calories significantly decreased peak d-βHB by ~ 1 mM (Figure 4A) and reduced the d-βHB AUC by 27% (*p* < 0.001, Figure 4B). There were no significant changes in d-βHB T_max_ (fed = 73 ± 6 min vs. fasted 66 ± 4 min). Despite the differences in d-βHB kinetics after the meal, there were no effects of food on urinary ketone excretion (Figure 4C), plasma AcAc (Figure 4D) or breath acetone (Figure 4E) following KE ingestion. Plasma AcAc kinetics followed a similar time course to d-βHB, with the ratio of blood d-βHB: AcAc being 6:1 when KE drinks were consumed whilst fasted, and 4:1 following the meal. As observed in Study 1, breath acetone concentrations rose more slowly than blood ketone concentrations, reaching a plateau at 150 min and remaining elevated for at least 4 h (Figure 4E).

The concentrations of blood d-βHB after KE drinks were highly repeatable whether consumed whilst fasted or fed (Figures 4F,G). The d-βHB C_max_ values ranged from 1.3 to 3.5 mM when fed and 2.3 to 4.7 mM when fasted. There was no significant effect of visit order on d-βHB kinetics, with the maximal difference in d-βHB C_max_ reached by one individual being 1.2 mM when fed and 1.9 mM when fasted. Approximately 61% of the variation in the data was attributable to feeding (fed vs. fasted), <1% to visit order, 16% to inter-participant variability, and the residual 24% variability due to non-specific random effects.

**Figure 5:**
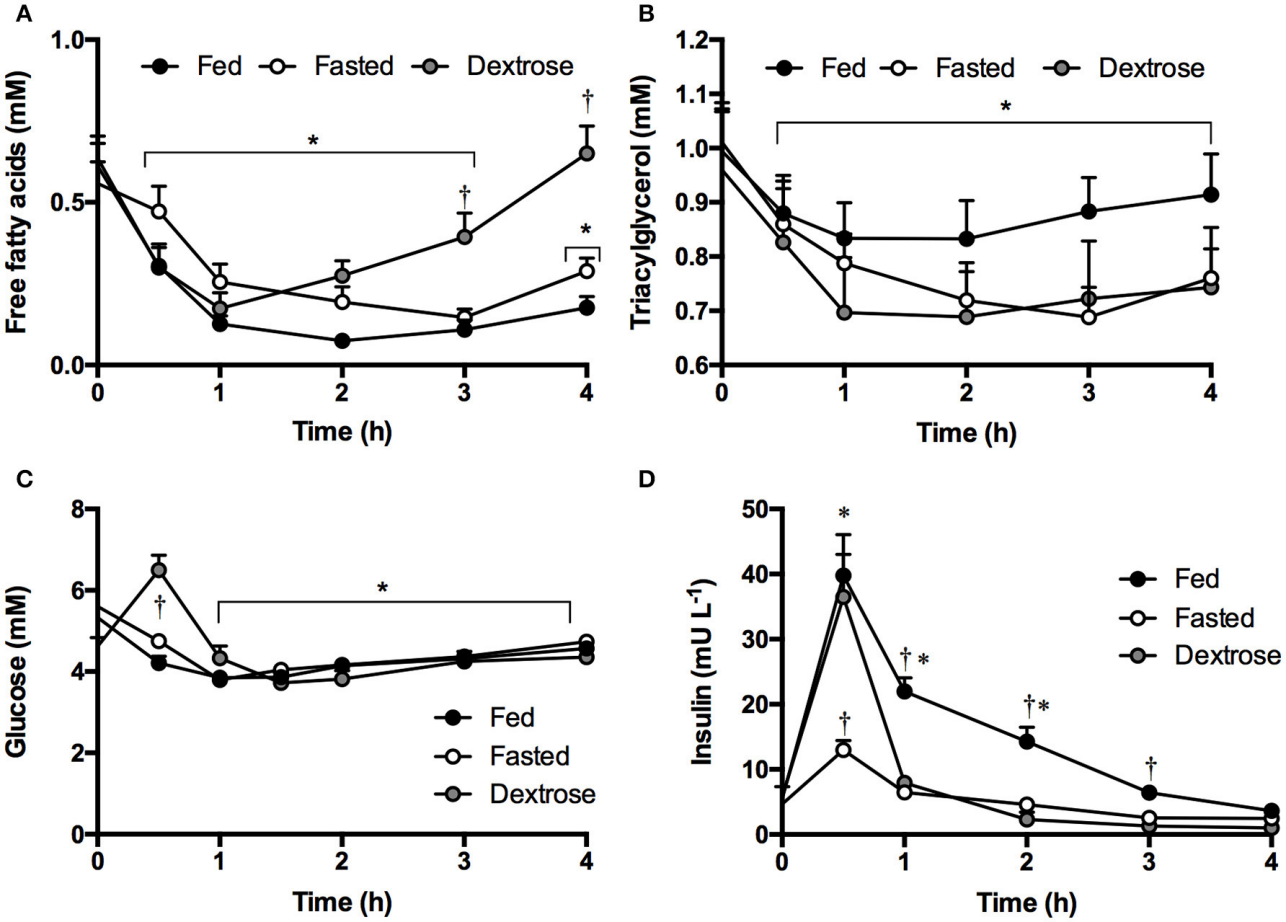
Plasma substrate concentrations following mole-matched ketone ester or isocaloric dextrose drinks in fed or fasted subjects (*n* = 16) at rest. Values are means ± SEM. **(A)** Plasma FFA. **(B)** Plasma TG. **(C)** Plasma glucose. **(D)** Plasma insulin. Significance denoted: ^*^p < 0.05 vs. baseline, ^†^*p* < 0.05 ketone vs. control.

KE consumption decreased FFA from 0.6 to 0.2 mM, TG from 1.0 to 0.8 mM, and glucose from 5.5 to 4.7 mM by the end of the study (4 h). The effect was not altered by a meal (Figures 5A–C). Dextrose drinks also lowered FFA from 0.6 to 0.2 mM and TG from 1.0 to 0.7 mM (Figures 5A, B). This was likely mediated by the transient increase in glucose, which rose from 4.6 to 6.5 mM following the dextrose drink (Figure 5C). The anti-lypoytic effect of dextrose drinks was shorter than that of KE drinks as d-βHB concentrations were elevated for longer after KE drinks than glucose after dextrose drinks. Insulin increased to ~ 35 mU.ml^−1^ after both the meal and the dextrose drink, but also increased to 13 ± 2 mU.ml^−1^ when KE was consumed whilst fasted owing to the 15 g of glucose in the flavored drink used as a diluent (Figure 5D).

### Effect of existing exogenous ketosis on KE uptake and elimination (Study 3)

**Figure 6:**
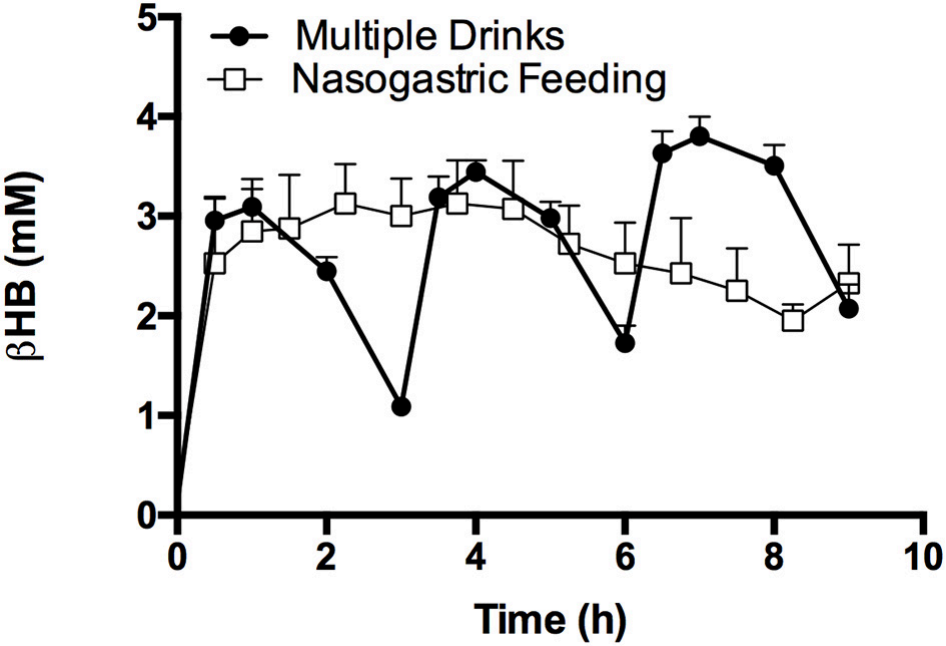
Blood d-βHB following 3 ketone ester drinks consumed following a fast, by subjects (*n* = 12), or with NG ketone ester feeding (*n* = 4); both methods maintained blood d-βHB concentrations above 1 mM (dotted line) for 9 h. Values are means ± SEM.

Serial drinks or a continuous NG infusion of KE effectively kept blood ketone concentrations >1 mM for 9 h (Figure 6). With drinks every 3 h, blood d-βHB rose and then fell, but had not returned to baseline (~ 0.1 mM) when the next drink was consumed. There was no significant difference in d-βHB C_max_ between drinks 2 and 3 (3.4 ± 0.2 mM vs. 3.8 ± 0.2 mM *p* = 0.3), as the rate of d-βHB appearance fell slightly with successive drinks (0.07 ± 0.01 mmol.min^−1^ and 0.06 ± 0.01 mmol.min^−1^ *p* = 0.6). d-βHB elimination was the same after each bolus (142 ± 37 mmol.min, 127 ± 45 mmol.min; and 122 ± 54 mmol.min). When KE was given via a nasogastric tube, the initial bolus raised blood d-βHB to 2.9 ± 0.5 mM after 1 h, thereafter continuous infusion maintained blood d-βHB between 2–3 mM. Total d-βHB appearance in the blood was identical for both methods of administration (Serial drinks AUC: 1,394 ± 64 mmol.min; NG infusion AUC: 1,305 ± 143 mmol.min. *p* = 0.6).

## Discussion

### Main findings

Exogenous ketones drinks are growing in popularity as a method to elevate blood ketone concentrations and mimic a ketogenic diet without the need for dietary changes (Ari et al., 2016; Cox et al., 2016; Kesl et al., 2016; Caminhotto et al., 2017; Evans et al., 2017). The present study describes the pharmacokinetic and pharmacodynamics properties of ketone ester and salt drinks in humans at rest, and characterizes the effects of a prior meal, which is pertinent to use as a dietary supplement. The main findings were that KE drinks elevated blood d-βHB > 50% higher than KS drinks, the latter significantly increasing blood l-βHB, which was metabolized more slowly by the body. Both drinks had similar effects on FFA, TG, glucose and electrolyte concentrations, although with disparate effects on pH. A prior meal decreased total blood d-βHB appearance after a KE drink. Finally, either three KE drinks or nasogastric feeding effectively maintained nutritional ketosis over 1 mM for 9 h.

### Comparative effects of ketone ester and salt drinks

The difference in peak blood d-βHB concentrations between matched amounts of βHB as ester or salts arose because the salt contained l-βHB, as the blood concentrations of d- plus l-βHB isoforms were similar for both compounds. It is unclear if kinetic parameters of KE and KS drinks would be similar if matched d-βHB were taken in the drinks. Unlike d-βHB, blood l-βHB remained elevated for at least 8 h following the drink, suggesting an overall lower rate of metabolism of l-βHB as urinary elimination of l-βHB was in proportion to plasma concentration. Despite similar concentrations of total βHB, breath acetone was ~50% lower following KS drinks compared to KE, suggesting fundamental differences in the metabolic fates of D- and L-βHB. These findings support both previous hypotheses (Veech and King, 2016) and experimental work in rats (Webber and Edmond, 1977), which suggested that the l-isoform was less readily oxidized than the d-isoform, and is processed via different pathways, perhaps in different cellular compartments. It seems that l-βHB is not a major oxidative fuel at rest, and may accumulate with repeated KS drinks. However, the putative signaling role of l-βHB in humans remains unclear. In rodent cardiomyocytes, l-βHB acts as a signal that modulates the metabolism of d-βHB and glucose, Tsai et al. (2006) although no differences in blood glucose were seen here. Furthermore, L-βHB can act as a cellular antioxidant, although to a lesser extent than D-βHB (Haces et al., 2008).

The effects of the two exogenous ketone drinks on acid-base balance and blood pH were disparate. In solution the ketone salt fully dissociates (giving a total of 3.2–6.4 g of inorganic cation per drink), allowing βHB^−^ to act as a conjugate base, mildly raising blood and urine pH, as seen during salt IV infusions (Balasse and Ooms, 1968; Balasse, 1979). Urinary pH increased with the salts as the kidneys excreted the excess cations. In contrast, KE hydrolysis in the gut provides βHB^−^ with butanediol, which subsequently underwent hepatic metabolism to form the complete keto-acid, thus briefly lowering blood pH to 7.31. Electrolyte shifts were similar for both KE and KS drinks and may have occurred due to βHB^−^ metabolism, causing cellular potassium influx and sodium efflux (Palmer, 2015).

### Ketone ester pharmacokinetics

As KE drinks achieved a significantly higher d-βHB concentrations than KS, we investigated factors that may be important in the use of ketone drinks to achieve nutritional ketosis. Initially we determined the repeatability of blood ketosis following KE drinks and found little variation in kinetic parameters between individuals. Variability between participants was less than within the population, and accurate individual prediction of the d-βHB C_max_ following a body-weight adjusted KE drink was achieved. Variability within individuals was likely due to normal daily changes in GI function, including gastric emptying, portal blood flow or intestinal transit time, which may alter KE hydrolysis and absorption.

As ketone drinks can deliver nutritional ketosis without fasting, we investigated the effect of food on KE uptake and metabolism. It is well documented that food in the gut can slow, or prevent, the uptake of small hydrophilic hydrocarbons, such as βHB (Melander, 1978; Toothaker and Welling, 1980; Horowitz et al., 1989; Fraser et al., 1995), so decreased gut βHB uptake is probably the cause of lower blood βHB following the meal. Despite higher blood βHB concentrations in the fasted state, the meal did not alter plasma AcAc. This suggests that the rate of conversion of βHB to AcAc may not match the rate of appearance of βHB following KE consumption. Alternatively, meal-induced changes in the hepatic ratio of NAD^+^:NADH may have altered the conversion of βHB to AcAc (Himwich et al., 1937; Desrochers et al., 1992).

As repeated KE consumption would be required to maintain nutritional ketosis, we investigated the kinetics of drinks in series and of continuous intra-gastric infusion. During starvation, the accumulation of ketones (>4 mM) reportedly inhibited ketone clearance from the blood, however the underlying mechanism is unknown (Hall et al., 1984; Wastney et al., 1984; Balasse and Fery, 1989). In Study 3, βHB uptake and elimination were identical for the second and third KE drinks, suggesting that βHB may have reached a pseudo-steady state should further identical boluses have been given at similar intervals. Furthermore, when the KE was given at a constant rate via a NG tube, blood ketone concentrations remained ~3 mM. Therefore, repeated KE drinks effectively maintain ketosis at the intervals and doses studied here.

### Effects of exogenous ketones on blood glucose, lipids and insulin

The metabolic phenotype of endogenous ketosis is characterized by lowered blood glucose and elevated FFA concentrations, whereas both blood glucose and FFA are lowered in exogenous ketosis. During endogenous ketosis, low insulin and elevated cortisol increase adipose tissue lipolysis, with hepatic FFA supply being a key determinant of ketogenesis. Ketone bodies exert negative feedback on their own production by reducing hepatic FFA supply through βHB-mediated agonism of the PUMA-G receptor in adipose tissue, which suppresses lipolysis (Taggart et al., 2005). Exogenous ketones from either intravenous infusions (Balasse and Ooms, 1968; Mikkelsen et al., 2015) or ketone drinks, as studied here, inhibit adipose tissue lipolysis by the same mechanism, making the co-existence of low FFA and high βHB unique to exogenous ketosis.

Blood glucose concentrations are decreased during both exogenous and endogenous ketosis, although by different mechanisms. During endogenous ketosis, dietary carbohydrate deficit is the underlying cause of low blood glucose, along with reduced hepatic gluconeogenesis and increased ketone production (Cahill et al., 1966). With exogenous ketosis, carbohydrate stores are plentiful, yet ketones appear to lower blood glucose through limiting hepatic gluconeogenesis and increasing peripheral glucose uptake (Mikkelsen et al., 2015). One clinical use of the ketogenic diet is to improve blood glucose control, yet the elevated blood FFA may increase the risk of heart failure (Holloway et al., 2009). Thus, the ability of exogenous ketones to lower blood glucose without elevating blood FFA concentrations could deliver the desired effect of the diet, whilst also decreasing a potential risk.

Interestingly, the effects of exogenous ketones on blood substrate concentrations were preserved with the metabolic stimulus of a mixed meal. Following KE drinks, FFA and glucose fell and remained low in both fed and fasted subjects, despite higher insulin throughout the fed arm, suggesting that there was no synergistic effect of insulin and βHB to further lower blood glucose or FFA. In agreement with previous work, the threshold for the effects of βHB on glucose and lipids appears to be low (<1 mM), as there was no significant dose-response relationship between increasing blood βHB and the small changes in plasma FFA, TG or glucose across all of the study drinks (Mikkelsen et al., 2015).

The effects of ketone drinks on endogenous insulin secretion are unclear. Whilst the small increase in plasma insulin after KE and KS drinks may have been due to the small quantity of dextrose in the diluent, it has been proposed that ketones could potentiate or even stimulate insulin secretion. Isolated pancreatic islets secreted insulin when stimulated by ketones at glucose concentrations of >5 mM (Biden and Taylor, 1983), and small amounts of insulin are secreted *in vivo* following exposure to exogenous ketones in animals (Madison et al., 1964; Miles et al., 1981). In response to an intra-venous 10 mM glucose clamp, ketone ester drinks increased glucose uptake and plasma insulin (Holdsworth et al., 2017). The increases in insulin with ketone drinks taken whilst fasted were small compared to the increases seen when the ketone ester drink was consumed with a meal and with consumption of a dextrose drink. Furthermore, the lack of difference in peak plasma insulin between the two latter conditions indicates that nutritional ketosis did not inhibit or increase normal carbohydrate induced insulin production.

### Monitoring exogenous ketosis

Given that blood βHB after identical ketone drinks can be affected by factors such as food or exercise (Cox et al., 2016), the accuracy of tools for non-invasive monitoring of ketosis should be investigated. Breath acetone and urinary ketone measurements provide methods to approximate blood ketosis without repeated blood sampling (Martin and Wick, 1943; Taboulet et al., 2007). However, breath acetone did not change as rapidly as blood βHB following KE and KS drinks. Acetone is a fat-soluble molecule, so may have been sequestered into lipids before being slowly released, resulting in the differences observed here. Similarly, significant differences in blood d-βHB between study conditions were not reflected in the urinary d-βHB elimination. As the amount of d-βHB excreted in the urine (≈0.1–0.5 g) represented ~1.5% of the total consumed (≈23.7 g), it appears that the major fate of exogenous d-βHB was oxidation in peripheral tissues. These results suggest that neither breath acetone nor urinary ketone measurements accurately reflect the rapid changes in blood ketone concentrations after ketone drinks, and that blood measurement should be the preferred method to quantitatively describe ketosis. That said, it should be noted that although commercial handheld monitors are the most practical and widely available tool for measuring blood ketones, they can overestimate blood D-βHB compared to laboratory measures (Guimont et al., 2015) and these monitors do not measure L-βHB and so may not provide accurate total blood ketone concentrations, especially if a racemic ketone salt has been consumed.

## Conclusion

In conclusion, drinks containing exogenous ketones, in either ester or salt form, can raise concentrations of blood βHB in humans, although elevation of l-βHB lasts longer after racemic KS consumption. Both KE and KS drinks mildly altered acid-base balance. Exogenous ketones lowered blood glucose and lipids without inhibiting endogenous insulin secretion. The KE delivered highly repeatable blood concentrations of d-βHB, although ketosis was decreased by a meal. Uptake and elimination of d-βHB were similar when several drinks were consumed in succession. The dietary KE could maintain ketosis using drinks taken regularly around a normal meal pattern, or using a continuous infusion via a nasogastric tube. Therefore, ketone drinks are a viable and practical alternative to dietary strategies to achieve ketosis.

## Ethics statement

The protocols carried out in these studies were approved by the the South West Frenchay NHS REC (15/SW/0244) (Study 1) and London Queen's Square REC (14/LO/0288) (Study 2 and 3). The studies were carried out in accordance with the recommendations of the Declaration of Helsinki, apart from pre-registration in a database. All subjects gave written informed consent in accordance with the Declaration of Helsinki.

## Author contributions

BS, KC, and PC designed the research studies. BS, PC, RE, SM, and PS carried out the studies. SH provided the gas analyser used in the study on behalf of NTT DOCOMO Inc. BS, MS, and SM analyzed the data and performed statistical analysis in collaboration with JM. BS wrote the paper with help from KC, PC, and OF. KC had primary responsibility for final content. All authors read and approved the final manuscript.

### Conflict of interest statement

Intellectual property covering uses of dietary ketone and ketone ester supplementation is owned by BTG Ltd., the University of Oxford, the National Institute of Health and TΔS Ltd. Should royalties ever accrue from these patents, KC and PC, as inventors, will receive a share of the royalties under the terms prescribed by the University of Oxford. KC is a director of TΔS Ltd., a company spun out of the University of Oxford to develop and commercialize products based on the science of ketone bodies in human nutrition. At the time of data collection and manuscript preparation, BS was an employee of TΔS Ltd., funded by the Royal Commission for the Exhibition of 1851. SH is an employee of NTT DOCOMO, Inc. (Japan). The other authors declare that the research was conducted in the absence of any commercial or financial relationships that could be construed as a potential conflict of interest.

## Abbreviations

AcAc: Acetoacetate
AUC: Area under the curve
βHB: β-hydroxybutyrate
d-βHB: d-β-hydroxybutyrate
l-βHB: l-β-hydroxybutyrate
BL: Baseline
C_max_: Maximal concentration
CV: Coefficient of variation
ELISA: Enzyme linked immunosorbent assay
FFA: Free fatty acids
GC-MS: Gas chromatography- mass spectrometry
KE: Ketone ester
KS: Ketone Salt
MCT: Monocarboxylate transporter
SEM: Standard error of the mean
TG: Triglyceride.
